# Unveiling the therapeutic potential of aromadendrin (AMD): a promising anti-inflammatory agent in the prevention of chronic diseases

**DOI:** 10.1007/s10787-025-01647-8

**Published:** 2025-02-11

**Authors:** Riham A. El-Shiekh, Mai Hussin Radi, Essam Abdel-Sattar

**Affiliations:** 1 Pharmacognosy Department, Faculty of Pharmacy, Cairo University, Kasr El-Aini Street, Cairo, 11562 Egypt; 2 Herbal Department, Egyptian Drug Authority (EDA), Giza, 12511 Egypt

**Keywords:** Aromadendrin, Dihydrokaempferol, Bioactivity, Pharmacological applications

## Abstract

In the dynamic realm of scientific inquiry, the identification and characterization of biologically active compounds derived from plant extracts have become of utmost significance. A particularly noteworthy flavonoid in this regard is aromadendrin (AMD), which can be found in a diverse range of foods, fruits, plants, and natural sources. The versatility of this compound is evident through its wide array of biological properties, including its well-documented anti-inflammatory, antioxidant, antidiabetic, neuroprotective, immunomodulatory, cardioprotective, and hepatoprotective effects. These diverse actions validate its potential utilization in addressing drug-related side effects, adverse reactions, neoplasms, ulcers, jaundice, diabetes mellitus, dermatitis, neurodegenerative diseases, cognitive disorders, polyploidy, carcinomas, common colds, and cumulative trauma disorders. This review aims to unlock the full potential of AMD and pave the way for groundbreaking advancements in the fields of medicine and nutrition. Prepare to embark on an enthralling journey as we unveil the hidden treasures and extraordinary prospects associated with AMD.

## Introduction

Flavonoids are found in large quantities in plants, including fruits, vegetables, nuts, and seeds. They are secondary metabolites of the polyphenol class. They have a significant and varied metabolic importance and are the source of plant pigments and colors. Because of this, flavonoids are considered a natural source of antioxidants and are consumed in the human diet (Bose et al. 2018; Mikail et al. 2022). The main structural feature of flavonoids that makes them physiologically and pharmacologically active is the presence of hydroxyl groups. The anti-inflammatory, antioxidant, anticarcinogenic, and antimutagenic properties of flavonoids have been demonstrated in vitro, in vivo, and in clinical investigations (Ahammed et al. 2018; Havsteen 2002; Mikail et al. 2022).

**Fig. 1:**
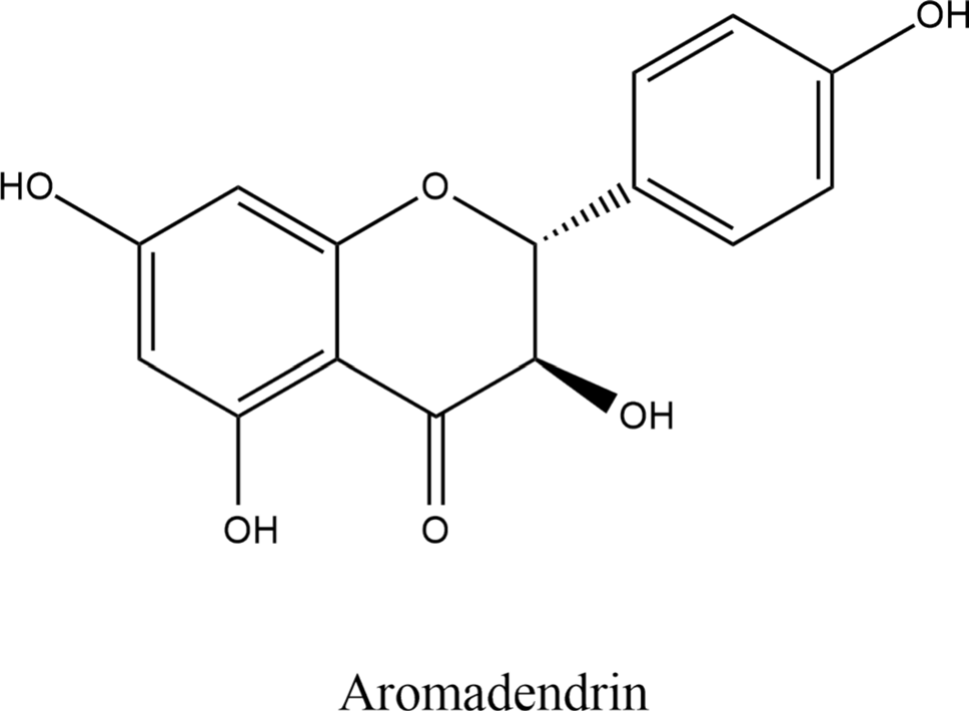
Chemical structure of the compound

The 15-carbon skeleton of flavonoids is made up of the heterocyclic ring C, which contains the embedded oxygen, and the two phenyl rings A and B. It is possible to shorten this carbon structure to C6-C3-C6 (Bose et al. 2018, de Souza Farias et al. 2021; Mikail et al. 2022). They fall into three groups: isoflavones, which are flavonoids linked in position 3 of the ring C; neoflavonoids, which are flavonoids linked in position 4; and additional subgroups based on the C ring's structural properties for flavonoids linked in position 2 of the ring C (Bose et al. 2018; De la Rosa et al. 2010; Lopez et al. 2001; Miyake et al. 2000; Tsao 2010). Flavones, flavonols, flavanones, flavanonols, flavanols or catechins, and anthocyanins are these subclasses. Finally, flavonoids with an open C ring are referred to as chalcones (De la Rosa et al. 2010; Han et al. 2007; López-Lázaro 2009; Manach et al. 2004; Reinli et al. 1996; Rouseff et al. 1987; Tsao 2010). AMD or dihydrokaempferol (C_15_H_12_O_6_) is a flavonoid subclassed to 2,3-dihydroflavonol isolated from acetone extracts of the wood of *Pinus sibirica* family *Pinaceae* (Lutskii et al. 1971) and the ethereal extract of *Persica vulgaris* fruit Family *Rosaceae* **(**Sadykov 1977**) **Fig. 1**.** It is functionally related to kaempferol. It is a conjugated acid of a ( +)-dihydrokaempferol 7-oxoanion (Reyes et al. 1923). AMD raised attention in previous years for researchers. In this review article, we attempt to provide an overview of the compound's evidence-based pharmacological activities, as well as its physical and chemical properties, synthesis, and quantification and quality control.

## Search strategy

A preliminary search using Google Scholar, Elsevier, Springer Nature, Wiley, PubMed, and EKB (Web of Science, Elsevier). The following Mesh items were created using research papers on "aromadendrin," "dihydrokaempferol," "biological activities," "natural sources," "biosynthesis," and "flavonoids". This search was performed using this **Synonyms:** Dihydrokaempferol, ( +)-Dihydrokaempferol, katuranin, Aromadedrin, ( +)-aromadendrin, Aromadendrol, 3,4',5,7-tetrahydroxyflavanone, (2R,3R)-2,3-Dihydro-3,5,7-trihydroxy-2-(4-hydroxyphenyl)-4H-1-benzopyran-4-one, (2R,3R)-3,5,7-Trihydroxy-2-(4-hydroxyphenyl) chroman-4-one, and (2R,3R)-3,5,7-Trihydroxy-2-(4-hydroxyphenyl)-2,3-dihydro-4H-chromen-4-one.

## Resources

The presence of AMD and its glycosides has been detected in a range of plant families, including Magnoliophyta, Pinophyta, and Pteridophyta. Within the Pteridophyta family, specific species such as Aspidiaceae, Aspleniaceae, Blechnaceae, Cyatheaceae, Dennstaedtiaceae, Equisetaceae, Ophioglossaceae, Polypodiaceae, and Schizaeaceae have been identified. Furthermore, AMD has been found in various foodstuffs, including dates, lovages, chicory roots, summer savories, shiitake mushrooms, pod mahogany, and Chinese fringe tree. Moreover, has been identified in various extracts, such as tea, broccoli, witch-hazel, propolis, and grapefruit. It has also been extracted from the ethanolic extracts of several bee species, including *Melipona*, *Tetragonisca*, and *Scaptotrigona* spp. (Singla et al. 2023).

## Physical and chemical properties

AMD CAS Registry Number®: 480-20-6 is a solid powder with the chemical formula C15H12O6. The boiling point is 638.00 to 639.00 °C at 760.00 mm Hg, while the melting point is 247–249 °C (Wishart et al. 2018, 2022, 2012, 2009, 2007). It has a molecular weight of 288.25 g/mol, a precise mass of 288.06338810 g/mol, four hydrogen bond donors, six hydrogen bond acceptors, and one rotatable bond (NCBI 2023).

Yellow amorphous powder (CH_3_OH); m.p. 59–60 °C; [α]D25 + 53° (c = 0.10, CH_3_OH).

## Spectroscopic analysis

**MS:** Negative FAB-MS* m*/*z* 287 [M – H]^–^; IR (KBr, v) 3,530, 1,660, 1,605, 1,550 cm^−1^.

**UV** (MeOH) λ_max_ (log ƹ): 214.0 (4.22), 293.0(4.01).

**IR** (KBr) *V*_max_, • cm^−l^: 3400 (OH), 1620 (C=O), 1520,1460 (aromatic ring), 1370, 1260, 1200, 1160, 1080, 1020,830.

^**1**^**H-NMR** (300 MHz, *methanol-d4*) 5 (ppm): 4.99 (1H,d, *J* = 11.7 Hz, H-2), 4.56 (1H, d, *J* = 11.7 Hz, H-3), 5.94 (1H, d, *J* = 2.1 Hz, H-8), 5.89 (1H, d, *J* = 2.1 Hz, H-6), 7.37 (2H, d, *J* = 8.4 Hz, H-2', 6'), 6.85 (2H, d, *J* = 8.4 Hz, H-3', 5') (Kim et al. 2020).

^**13**^**C-NMR** (75 MHz, *methanol-d4*) 6 (ppm): 84.0 (C-2), 72.6 (C-3), 197.5 (C-4), 164.3 (C-5), 96.3 (C-6),167.7 (C-F), 95.3 (C-8), 163.5 (C-9), 100.8 (C-10), 128.3 (C-1'), 129.4 (C-2', 6'), 115.1 (C-3', 5'), 158.2 (C-4') (Zhang et al. 2006).

## Quantification and quality control

Kim et al. (2017) used HPLC to quantitatively analyze the butanol and ethyl acetate extracts of *Brugmansia arborea* L. Flowers. AMD was assigned to the ethyl acetate fraction at 365 nm with a retention duration of 25.8 min, a regression curve of 277.56x + 2125.6, and an R_2_ of 0.997. The trial yielded an AMD of 12.7 ± 0.7%. The method for analyzing flavonoids in B. arborea flowers used HPLC (Alliance e2690; Waters) paired with photodiode array detector (PDA 2998; Waters) and column (Agilent, USA) Poroshell 120 SB-C18 (dimensions: 120 Å, 2.7 μm, 4.6 × 150 mm). The oven temperature is 30°C, the injection volume is 5 μl, and the wavelength used is 365 nm. Solvents A (H_2_O: formic acid = 99.9:0.1, *v/v*) and B (acetonitrile) were utilized in the mobile phase (0.8 ml/min) in a gradient manner (Table 1). The regression curve employed various concentrations (250, 125, 50, 25, and 12.5 μg/ml) for the stock solution of the produced sample and the AMD standard at 1mg/ml in methanol (Kim et al. 2020).

**Table 1:** Gradient system used in analytical quantification of flavonoids in *B. arborea* flowers (Kim et al. 2020)

Solvent A%	Solvent B%	Time
92%	8%	0–2 min
88%	12%	3 min
84%	16%	4–12 min
80%	20%	15–18 min
76%	24%	21 min
70%	30%	22–26 min
50%	50%	30 min
20%	80%	33 min
92%	8%	34–36 min

The chemical and biological evaluation of geopropolis from Melipona orbignyi, stingless bee, was conducted using the high-performance liquid chromatography coupled to a diode array detector and mass spectrometry (HPLC–DAD-MS) method. The preparation of the hydroalcoholic extract of geopropolis (HEGP) was done, and 1 mg/mL was injected into the ultra-fast liquid chromatograph (UFLC) (LC-20AD, Shimadzu) along with a mass spectrometer that used electrospray ionization (ESI) and a diode array detector that monitored the sample between 240 and 800 nm. The analyzer (micrOTOF-Q III, Bruker Daltonics) monitored the sample between m/z120 and 1200 in both positive and negative ion mode, and the column's dimensions were (Kinetex, 150 × 2.2 mm id, 2.6μm). The injection volume was 1 μL, the flowrate was 0.3 mL/min, and the oven temperature was 50◦C. The mobile phase consisted of deionized water (A) and acetonitrile (B) in a gradient system with 3% B (0–8 min), 3–25% B (8–30 min), and 25–80% B (30–60 min), both containing 0.1% formic acid (*v/v*). Retention duration of 25.2 min, UV of 289 nm, [M-H] − 287.0553 m/z, error of 2.7 ppm, and MS/MS equal to 259 *m/z* (C_14_H_11_O_5_) − , 177 (C_10_H_9_O_3_) were the times this AMD peak emerged (Santos et al. 2017).

In a recent study, the phenolic content of eight different types of stingless bee honey (Meliponinae) from southern Brazil was investigated. AMD was one of the phenolic components that were identified and quantified using an HPLC system in conjunction with a mass spectrometry system that included a hybrid triple quadrupole/linear ion trap mass spectrometer, the Q TRAP 200 (LC-ESI–MS/MS-Applied Biosystems/MDS Sciex, Concord, Canada). To achieve the chromatographic separation, a gradient of (A) 95% methanol in water and (B) 0.1% formic acid in water was employed as the mobile phase. Phenolic compounds were separated at 30 °C on a Synergi column (4.6 μm particle size, 150 mm, 2.0 mm). The gradient elution system was 0–5 min, 10% A; 5–7 min, 90% A; 7–10 min, 90% A; and 10–17 min, 10% A. The HPLC system was linked to the mass spectrometry system, which comprised a hybrid triple quadrupole/linear ion trap mass spectrometer Q TRAP 3200 (Applied Biosystems/MDS Sciex, Concord, Canada). Software Analyst version 1.6.2 was used for the LC–ESI–MS/MS data analysis and system control. The Turbo Ion Spray TM source (electrospray-ESI) was used in negative ion mode for the studies (Biluca et al. 2020). This study developed the total ion chromatogram (TIC) of all phenolic chemicals, including AMD (30), in a solvent at the calibration curve's medium concentration level. Table 2 displays the parent and quantitative ions, retention durations, limits of detection (LOD), and quantification (LOQ) of AMD found in honey produced by stingless bees.

**Table 2:** Parent and quantitative ion, retention times, limits of detection (LOD) and quantification (LOQ) of AMD identified in stingless bee honey (Biluca et al. 2020)

Property	AMD
Quantitative ion (m/z) Q1	286.824
Quantitative ion (m/z) Q3	123.90
Retention time (min.)	11.29
LOD (mg L ^−1^)	0.002
LOQ (mg L ^−1^)	0.007

## Chemical biosynthesis

**Fig. 2:**
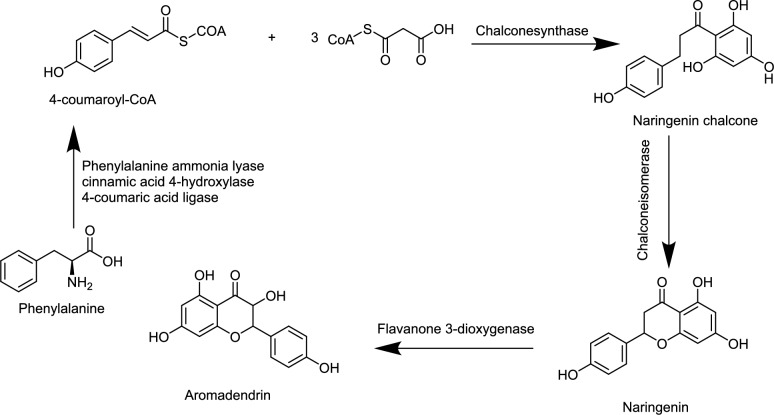
Biosynthesis of aromadendrin (AMD) (Abdel-Sattar et al. 2000; Ali et al. 2023)

A biosynthetic route for AMD was illustrated in Fig. 2**.**

## Pharmacological activities

### Anti-tyrosinase activity

The researchers' investigation focused on isolating tyrosinase inhibitors from crude extracts of Manilkara zapota bark based on bioactivity. AMD had the highest anti-tyrosinase activity of any of the substances examined. Comparative research found that AMD inhibited monophenolase more effectively than kojic acid, a well-known tyrosinase inhibitor. Furthermore, it displayed diphenolase activity comparable to kojic acid. The inclusion of particular hydroxyl groups in its structure helps to explain its exceptional action. These hydroxyl alterations influence tyrosinase function by forming hydrogen bonds and hydrophobic interactions. Further research is needed to unravel the underlying mechanisms and investigate the potential applications of AMD as a therapeutic agent targeting tyrosinase-related illnesses (Chunhakant and Chaicharoenpong 2019).

### Antioxidant activity

While investigating the radical scavenging properties of fractions and flavonoids extracted from B. arborea flowers. Kim et al. discovered that AMD exhibited the maximum antioxidant activity, owing to the presence of four hydroxyl groups. AMD has a determined antioxidant capacity (mg vitamin C equivalents/100 mg) of 22.6 ± 1.1b for DPPH and 155.6 ± 2.5b for ABTS (*p* < 0.05) (Kim et al. 2020). In an early investigation, kwon et al. reported that AMD has a modest in vitro antioxidant activity with IC50 equates to > 1000 μM for flavonoids aglycones and glycosides (Kwon et al. 2004).

### Anti-inflammatory activity

Lee et al. (2013) sought to determine the precise mechanism of AMD as an anti-inflammatory drug. The study looked at how it affected RAW 264.7 macrophage cells that had been activated with lipopolysaccharide (LPS). AMD decreased the production of proinflammatory cytokines NO and PGE2 in LPS-stimulated RAW 264.7 macrophage cells. The cells were treated with AMD (10, 50, 100, or 200 μM) for 1 h before receiving LPS (200 ng/ml). AMD dramatically reduced NO and PGE2 release in LPS-stimulated RAW 264.7 cells in a concentration-dependent manner. However, it has a weaker inhibitory impact than the usual flavonoids wogonin and quercetin. The inhibition's mechanism was explored to see whether it was related to the downregulation of iNOS and COX-2 expression. Treatment with AMD dramatically inhibited LPS-induced iNOS and COX-2 overexpression in a concentration-dependent manner. Consistent with the decrease in protein levels, AMD significantly reduced LPS-induced up-regulation of iNOS and COX-2 mRNA expression, suggesting that AMD reduces NO and PGE2 emissions via decreasing expression of the relevant genes. AMD's capacity to inhibit LPS-induced κB degradation was studied. LPS therapy drastically lowers κB levels. AMD's capacity to prevent nuclear translocation of the NF-κB p65 subunit was studied. LPS treatment significantly increased NF-κB's nuclear translocation. AMD inhibited LPS-induced nuclear translocation of NF-κB in a concentration-dependent manner.

The study looked at how AMD influenced the phosphorylation of JNK, ERK, and p38 kinases in RAW 264.7 macrophages in response to LPS. After a 30-min pretreatment with AMD (10, 50, 100, and 200 μM), the cells received a 30-min LPS treatment (200 ng/ml). In RAW 264.7 cells, AMD significantly inhibited JNK phosphorylation induced by LPS in a concentration-dependent manner. In contrast, AMD did not significantly reduce ERK or p38 phosphorylation. Based on the research findings, it is strongly suggested that the LPS-induced inflammatory response in RAW 264.7 cells is mediated by JNK signaling (Lee et al. 2013).

JIN-MI et al. (Park et al. 2023) conducted a novel investigation to evaluate the therapeutic effects of AMD on the development of allergic asthma in vitro and in vivo. In A549 airway epithelial cells, phorbol 12-myristate 13-acetate (PMA, 100 nM) was used to induce inflammation. A549 and eosinophil EOL-1 cell cohesion were studied. Alum (3 mg) and Ovalbumin (30 or 60 μg) were designed to cause allergic asthma in mice. Mice were administered 5 or 10 mg/kg, p.o. AMD to determine if it may inhibit the development of allergic asthma. Hematoxylin and eosin/periodic acid Schiff staining, enzyme-linked immunosorbent assay, and western blotting were utilised to study AMD's therapeutic effect on bronchial inflammation. Pretreatment of A549 cells with AMD Successfully reduced cytokine (IL-1β/IL-6/TNFα/MCP-1) and NF-κB activation in response to PMA. The data is presented as mean ± SD (#*p* < 0.01 for comparison with control; *p < 0.05, **p < 0.01 for comparison with PMA). Furthermore, AMD reduced the adherence of A549 cells and eosinophils. After one hour of AMD incubation, A549 cells were kept alive for five hours using PMA. Following that, the labelled EOL-1 cells were placed in a culture plate with A549 cells and cultured for one hour. The cells were then viewed and analyzed using a fluorescence microscope.

In mice with ovalbumin-induced allergic asthma, AMD reduced the elevation of various cell types, including eosinophils, Th2 cytokines, MCP-1 in bronchoalveolar lavage fluid, IgE in serum, and inducible nitric oxide synthase/cyclooxygenase-2 expression in lung tissue. Data are shown as mean ± SD (*p < 0.05 for comparison with the OVA group and #*p* < 0.05 for comparison with the normal control). ARO 5: 5 mg/kg AMD (ARO)-treated OVA mice; OVA: OVA-sensitized mice; DEX: 1 mg/kg DEX-treated OVA mice; ARO 10: 10 mg/kg ARO-treated OVA mice. Histological studies found that AMD reduced cell inflow and mucus development in allergic asthmatic mice's lungs (Ovalbumin group vs. NC, *p* < 0.05), while ARO and DEX impeded this process. The OVA + 10 mg/kg ARO group outperformed the ovalbumin and oval albumin groups (*p* < 0.05), while the ovalbumin group outperformed the OVA + 1 mg/kg DEX group.

In mice with allergic asthma, AMD reduced NF-κB activation in their lungs. AA mice had significantly higher levels of NF-κB and IκB in their lung tissue lysates (*p* < 0.05) than the OVA group. ARO and DEX inhibited NF-κB p65 and IκB activation in the lungs of AA mice (ovalbumin group vs. OVA + 1 mg/kg DEX group, *p* < 0.05; ovalbumin group vs. OVA + 5 mg/kg ARO group, *p* < 0.05; ovalbumin group vs. OVA + 10 mg/kg ARO group). ARO's positive benefits may be attributed to its ability to modulate NF-κB activity. The study suggests that ARO may be a highly effective adjuvant for the treatment of AA due to its modulation of bronchial inflammation (Park et al. 2023).

Kim et al. studied the anti-inflammatory potential of Brugmansia arborea L. flower fractions and isolated flavonoids. They tested the inhibition strength of fractions and compounds (kaempferin, kaempferitrin, kaempferol 3-O-β-d-glucopyranosyl-7-O-α-l-rhamnopyranoside, quercetin 3,7-di-O-α-l-rhamnopyranoside, and AMD) on NO production in RAW 264.7 cells induced by LPS. AMD inhibited NO production less than other flavonoids (*p*< 0.05). Fractions and flavonoids were also investigated for their inhibitory effects on iNOS and COX-2 production. Western blotting was used to quantify inhibition activity against the standard β-actin. All fractions and substances significantly reduced iNOS protein expression produced by LPS treatment, although Kaempferin was the most effective (*p* < 0.05). AMD has a minimal effect on the LPS-induced rise in COX-2 protein synthesis (Kim et al. 2020).

In a recent study, AMD with another 10 phenolic compounds isolated from *E. africana* leaves hydroalcoholic extract were screened for their in vitro anti-inflammatory activities. It was found out that AMD influences inhibition of IL-6 with MI (%) 67.0 ± 2.6 and IC_50_ 6 μM 95% CI $\left[4.1-88\right]$ (Codo Toafode et al. 2022).

Another study looked at the leaves of Olea europaea cultivar Nocellara del Belice, and one of the extracted compounds was AMD, which was tested for anti-inflammatory activity in normal keratinocytes. AMD has been shown to alter the expression of ICAM-1, MCP-1, and IL-8 in NCTC 2544 cells stimulated by histamine and IFN-γ. AMD substantially inhibited IFN-γ and histamine-induced MCP-1 production, with a 52% suppression at a low dose (10 μg mL-1) and 59% at a larger dose (100 μg mL^–1^). In addition, histamine and IFN-γ both potently increased IL-8, which AMD significantly reduced in a dose-dependent manner. Results are the mean ± SEM. *p < 0.05, **p < 0.01 compared with untreated control suggesting that AMD could be the active compound in *O. europaea* extract (Venditti et al. 2013).

An in vitro Bio-guided investigation of Populus davidiana stem bark revealed that the methanolic extract inhibits cyclooxygenase (COX-l, COX-2). The phytochemical analysis led to the identification of ten flavonoids, including AMD. The extracted compounds were evaluated against COX-l and COX-2. Kaempferol strongly inhibited COX-1 (IC_50_ = 7.5 μM) and had a moderate effect on COX-2 (IC_50_ = 269.2 μM). AMD showed considerable inhibition against COX-1 alone (IC_50_ = 257.7 μM). AMD had a modest effect on enzyme xanthine oxidase on superoxide radical scavenging (IC_50_ = 69.9 μM) and an effect on xanthine oxidase (IC_50_ > 200 μM) (Zhang et al. 2006).

### Acute pancreatitis

To investigate the possible therapeutic benefits of AMD, animal models of acute pancreatitis produced by caerulein and lipopolysaccharide (LPS) were used. H&E staining demonstrated severe pancreatitis-related characteristics, such as pancreatic cell expansion and parenchymal necrosis, when caerulein and LPS were injected. However, therapy with AMD at dosages of 40 and 80 mg/kg dramatically improved these pathological features. The acute pancreatitis group also had a significantly higher pancreatic weight-to-body weight (PW/BW) ratio and blood levels of amylase and lipase. Notably, treatment of AMD at doses of 40 and 80 mg/kg resulted in a significant decrease in these values, showing that AMD successfully reduced pancreatic damage. AMD treatment at 40 and 80 mg/kg doses significantly reduced IL-1, IL-6, and TNF-α concentrations in blood and pancreatic tissue mRNA expression when compared to the acute pancreatitis group. It is worth mentioning that when AMD was given alone at a dose of 80 mg/kg, there were no significant changes in cytokine levels. These data indicate that rather than preventing oxidative stress-induced cytokine increase, AMD actually lowered their levels (Liang et al. 2020). In addition to its therapeutic actions in acute pancreatitis models, AMD has been shown to have antioxidant properties. Previous investigations isolated AMD from Riesling wine and demonstrated its antioxidant effects (Baderschneider and Winterhalter 2001). Another study extracted AMD from *Dioon spinulosum* leaves and discovered that it had the strongest antioxidant activity (89.6% inhibition), outperforming amentoflavone and sotusflavone (with inhibition rates of 79.1% and 64.3%, respectively), which served as positive controls (Negm et al. 2021).

### Antidiabetic activity

Zhang, Lee, and colleagues studied AMD from Gleditsia sinensis Lam for its ability to stimulate glucose absorption and improve insulin resistance, as well as the molecular processes underlying these effects. AMD's capacity to stimulate 2-NBDG absorption in HepG2 cells and differentiate 3T3-L1 adipocytes was tested. This is to study the impact on glucose uptake. Rosiglitazone was used as a positive control. AMD (30 µmol/l) significantly increased 2-NBDG absorption in HepG2 cells (*p* < 0.01). AMD significantly boosted insulin-stimulated 2-NBDG absorption in 3T3-L1 adipocytes at 3 µmol/l (*p* < 0.05) and 30 µmol/l (*p* < 0.01). Treatment with 30 µmol/l AMD increased insulin-stimulated 2-NBDG absorption in both cell types compared to 30 µmol/l rosiglitazone. Thus, AMD increases insulin-induced glucose absorption.

The study also examined 3T3-L1 preadipocytes that had been differentiated into adipocytes and stained with Oil Red O to assess the accumulation of intracellular lipid droplets to compare the adipogenic properties of AMD and rosiglitazone. The measurement demonstrated that 30 µmol/l rosiglitazone or AMD treatment enhanced lipid accumulation by around 2.4 and 2.0 times, respectively. Treatment with 30 µmol/l AMD increased adipocyte lipid content less than treatment with 30 µmol/l rosiglitazone. RT-PCR was performed to analyse the impact of AMD on PPARγ2 and aP2 mRNA expression in differentiated 3T3-L1 adipocytes. Treatment with rosiglitazone and AMD increased aP2 mRNA expression by 2.3- and 2.8-fold after 3 and 30 µmol/L rosiglitazone, and 1.7- and 2.7-fold after 3 and 30 µmol/L AMD, respectively. RT-PCR data showed that 30 µmol/l AMD dramatically increased PPARγ2 protein levels in differentiated adipocytes compared to the same amount of rosiglitazone. In addition, insulin-mediated Akt/PKB phosphorylation was assessed in high glucose-induced, insulin-resistant HepG2 cells to determine AMD's ability to restore insulin-stimulated Akt/PKB signalling in insulin-resistant cells. In high-glucose circumstances, treatment with 3 µmol/l or 30 µmol/l rosiglitazone significantly increased Akt/PKB phosphorylation. While 3 µmol/l AMD did not raise Akt/PKB phosphorylation, 30 µmol/l AMD under high-glucose circumstances increased insulin-stimulated Akt/PKB phosphorylation to a level equal to 30 µmol/l rosiglitazone. In conclusion, AMD is a potential agent for the improvement of insulin-resistant sensitivity to insulin through Akt/PKB phosphorylation pathway (Zhang et al. 2011).

While examining prickly pears (*Opuntia ficus*-indica), which have been grown as a crop in semiarid and arid regions around the world. The efficacy of prickly pear root extracts in managing diabetes and its complications was evaluated using a variety of in vitro bioassays. The ethyl acetate-soluble fraction inhibited rat lens aldose reductase activity, advanced glycation end products, and α-glucosidase activity, making it the most effective in preventing diabetes and its repercussions, according to bioguided assessment. Additionally, pure AMD from prickly pear root was tested for its potential to inhibit aldose reductase and the development of advanced glycation end products (AGEs) and shown to be effective. At three distinct dosages (0.03, 0.06, and 0.012 µg/mL), the percentage inhibition of aldose reductase was 42.39, 47.76, and 63.64, respectively. The IC_50_ is 0.065 ± 0.005 µg/mL. At concentrations of 0.5, 1, and 2 µg/mL, the percentage inhibition of AGE production is 2.09, 12.35, and 20.69, respectively. IC_50_ = 17.7 ± 3.35 µg/mL (Jeon et al. 2011).

### Neuroprotective activity

When Hyun-Su et al. researched the neuroprotective activity of AMD extracted from Chionanthus retusus flowers, they discovered that it protects neuronal cells from methamphetamine-induced neurotoxicity and investigated its mechanism of action. They first investigated whether AMD treatment was damaging to SH-SY5y neuronal cells by utilising the methyl thiazol tetrazolium (MTT) assay to assess cell survival. AMD dosages of up to 40 µM did not result in cytotoxicity. An AnnexinV/propidium Iodide (PI) experiment was conducted to determine whether AMD induced an apoptotic pathway in SH-SY5y cells. AMD concentration (0–40 µM) did not affect apoptosis in SH-SY5y cells, showing it is non-cytotoxic. It was studied whether AMD pre-treatment impacts METH-induced ER stress to better understand the underlying mechanism of AMD's protective effect against METH-induced neuronal cytotoxicity. C/EBPβ and CHOP mRNA levels were identified as indications of ER stress. Pre-treatment with AMD reduced C/EBPβ mRNA expression in a time- (*df* = 11, *p* < 0.05) and dose-dependent (*df* = 17, *p* < 0.05) manner compared with mock-treated cells. AMD lowered CHOP mRNA levels in METH-exposed neuronal cells (*df* = 11, *p* < 0.05 vs METH-treated and *df* = 17, *p* < 0.05 vs mock-treated). Western blotting revealed that pre-treatment with AMD reduced the expression of ER stress indicators at the protein level. METH-induced cytotoxicity suppresses the phosphorylation of PI3K/Akt/mTOR, hence the study investigated whether AMD is linked to this pathway in METH-exposed neurons. METH exposure reduces PI3K phosphorylation, but AMD pre-treatment enhances PI3K phosphorylation in SH-SY5y cells (*df* = 11, *p* < 0.05 compared to METH-treated). Pre-treatment with AMD in a METH-exposed environment maintained downstream PI3K signaling pathways, including Akt (*df* = 11, *p* < 0.05) and mTOR (*df* = 11, *p* < 0.05 compared to METH-treated). According to the findings, AMD helps METH-exposed neuronal cells survive by restoring phosphorylation of the PI3K/Akt/mTOR signaling pathway. METH-induced neurotoxicity includes a decrease in the phosphorylation of the PI3K/Akt/mTOR pathway, which is a key sign of autophagy.

METH treatment raises Beclin1 mRNA levels, a key autophagic pathway marker, in a dose- and time-dependent manner (*df* = 11, *p* < 0.05 compared to mock-treated). In addition, they investigated whether AMD pretreatment affects the autophagic route induced by METH. In SH-SY5y cells, pre-treatment with AMD substantially suppressed the increased mRNA levels of Beclin1 and LC3 following METH exposure (*df* = 11, *p* < 0.05 against METH-treated cells). At the protein level, AMD suppressed METH-induced autophagy (*df* = 11, *p* < 0.05 versus METH treated). In addition, the IncuCyte® imaging system was used to determine the intensity of Annexin V in SH-SY5y cells. The intensity of AnnexinV in SH-SY5y cells increased when exposed to 2 mM METH. However, AMD pre-treatment attenuated the impact in a dose-dependent manner (*df* = 17, *p* < 0.05 compared to METH-treated). AMD pre-treatment reduces the number of AnnexinV/PI-positive cells, as demonstrated by the AnnexinV/PI apoptosis assay (*df* = 11, *p* < 0.05 compared to METH-treated). These findings demonstrate that pre-treatment with AMD partially reduces METH-induced apoptosis in SH-SY5y neuronal cells.

Western blot analysis was utilised to measure the expression levels of the caspase family of proteins and Bax to determine whether AMD pre-treatment affects the expression of apoptosis-related proteins under METH exposure. METH reduced the expression of the anti-apoptotic proteins Bcl-2, Caspase 3, and Caspase 7, while increasing the expression of Bax, which activates the apoptotic pathway in SH-SY5y cells. In METH-exposed SH-SY5y cells, AMD pre-treatment raised Bcl-2, Caspase3, and Caspase7 expression but decreased Bax expression (*df* = 11, *p* < 0.05 compared to METH-treated). The IncuCyte® imaging system's caspase3/7 fluorescence detection showed that AMD pre-treatment maintains the dose-dependent reduction in Caspase3/7 intensity generated by METH exposure (*df* = 17, *p* < 0.05 compared to METH-treated). These results suggest that, in the presence of METH exposure, pre-treatment with AMD moderately restores anti-apoptotic protein expression while decreasing pro-apoptotic protein production. In contrast, under METH-exposed conditions, the mTOR signaling pathway is essential for controlling cell fate in autophagic or apoptotic pathways. An inhibitor experiment was conducted using rapamycin, a strong inhibitor of mTOR phosphorylation, to investigate whether pretreatment with AMD had a protective effect against METH-induced neurotoxicity via the mTOR signaling pathway. Pre-treatment with rapamycin in METH-exposed situations modulates AMD's boosting effect on mTOR phosphorylation (*df* = 11, *p* < 0.05 between two groups). Pre-treatment with rapamycin abolished AMD's anti-autophagic action in METH-exposed situations (*df* = 11, *p* < 0.05 across two groups). Pre-treatment with rapamycin significantly reduced AMD's anti-apoptotic activity in METH-exposed conditions (*df* = 11, *p* < 0.05 between the two groups). The IncuCyte® imaging system detected Annexin V fluorescence and found that rapamycin pre-treatment diminished AMD protection under METH exposure (*df* = 11, *p* < 0.05 between the two groups). These data imply that AMD-induced mTOR phosphorylation inhibits autophagy and death in neural cells exposed to METH (Lee et al. 2021).

Yanxian Zhang et colleagues. discovered that repurposing AMD as a dual amyloid promoter reduced neuroblastoma/insulinoma toxicity of both hIAPP37 (related with T2D) and Aβ42 (associated with AD), while also accelerating amyloid aggregation/fibrillization. The addition of AMD to amyloid solutions with 1 to 5 molar ratios significantly accelerated the fibrillization of Aβ42 by 86–114% and hIAPP by 20–68%. This was demonstrated by the growth phase being promoted, the lag phase being shortened or bypassed, and the amyloid species being rapidly converted into higher ordered β-structure-rich aggregates, according to ThT, AFM, and CD results. Additional seeding tests revealed that, while AMD does not accelerate the fibrillization of higher-order protofibrils, it is more effective at hastening the aggregation and structural conversion of amyloid species during the lag and early development phases. Furthermore, MTT and LDH cell assays showed that AMD-treated cell samples protected cells against toxicity caused by Aβ and hIAPP. Cell apoptosis decreased by 45–67% (hIAPP), while cell viability increased by 12–15% (Aβ) and 10–49% (hIAPP). LUV tests revealed AMD's protective action in cell toxicity, with the reduction in membrane leakage related to the suppression of harmful oligomer formation. This study proposes repurposing AMD as an amyloid promoter, rather than an inhibitor, to accelerate amyloid production and reduce toxicity for both Aβ and hIAPP. Furthermore, AMD's sequence-independent promoting impact could be applied to additional amyloid proteins (Zhang et al. 2020).

### Immunomodulatory activity

Hyun-su et al. investigated the ability of the flavonoid AMD to reduce T cell activity in order to develop a non-cytotoxic immunosuppressive medication. Western blotting, flow cytometry, conventional and qualitative PCR, and MTT tests were used to investigate the effects of AMD on activated T cell activity, cell survival and confluence, and proximal signal transduction. AMD effectively regulated IFNγ and IL-2 production from activated Jurkat T cells in vitro without damage. Additionally, AMD therapy lowered the expression levels of surface molecules CD69, CD25, and CD40L. Activated T cells pre-treated with AMD exhibited reduced calcium (Ca^2+^) influx. AMD decreased NFAT cell dephosphorylation and nuclear translocation, as shown by Western blotting. The NF-κB and MAPK pathways play a role in inhibiting AMD. The findings revealed that AMD reduces T cell activation via modulating Ca2 + influx and inhibiting NFAT activity in activated T cells (Lee and Jeong 2020).

### Hepatoprotective activity

Zhihui Zhou looked at the mechanism of action and protective role of AMD in mice with septic liver injury. AMD (SMB00175, Sigma-Aldrich) was administered intraperitoneally (by hypodermic needle) to eight-week-old male C57BL/6 mice (*n* = 6 per group). H&E stains were used to analyze histological structural changes in the liver, while DAPI/Tunnel staining was used to measure liver cell apoptosis. TNF-α, IL-1β, and IL-6 mRNA expression levels were measured by qRT-PCR. The enzyme-linked immunosorbent assay (ELISA) was used to measure TNF-α, IL-1β, and IL-6 levels, as well as antioxidant activities such as glutathione (GSH), superoxide dismutase (SOD), catalase (CAT), and malondialdehyde (MDA). Western blotting was used to assess the amounts of p65, p-p65, p-IκBα, and IκBα proteins. AMD therapy resulted in substantial structural damage to liver tissues, including edema, necrosis, and neutrophil infiltration (*p* < 0.05). AMD reduced increased blood levels of alanine aminotransferase (ALT) and aspartate aminotransferase (AST) in CLP mice. It also reduces liver injury and cell death. AMD significantly lowered TNF-α, IL-1β, and IL-6 levels in mice, as well as the effects of GSH and antioxidant enzymes (SOD and CAT) (p < 0.05). AMD reduced the phosphorylation of IκBα and p65 (*p* < 0.05) and prevented MDA levels from rising. By blocking NF-κB signaling in vivo, AMD shields the liver of mice from damage caused by sepsis, indicating a possible treatment approach for sepsis-induced liver damage (Zhou and Yin 2022).

In this study, AMD was investigated as a potential intervention for CCl_4_-induced liver injury and hepatic fibrosis in a mouse model. In vitro experiments were conducted to examine the expression levels of PARP-1-related proteins and phosphorylation. Molecular docking and molecular dynamics platforms were used to analyze the binding pattern between AMD and PARP-1. The results demonstrated that AMD effectively reduced CCl_4_-induced liver injury and fibrosis in mice. In addition, it also exhibited protective effects against CCl_4_-induced toxicity in HepG2 cells and inhibited the synthesis of α-SMA and Collagen 1/3 in LX-2 cells. Molecular docking analysis revealed that AMD competitively bound to Glu-988 and His-862 residues of PARP-1, a DNA repair enzyme located upstream, which resulted in moderate inhibition of its overactivation. This led to the maintenance of NAD^+^ levels and energy metabolism in hepatocytes, as well as the inhibition of PARP-1-regulated downstream signaling pathways (such as TGF-β1), related proteins (such as p-Smd2/3), and inflammatory mediators. Overall, AMD attenuated CCl_4_-induced liver injury and hepatic fibrosis in mice through a different mechanism compared to other reported flavonoids. Its action involved competitive binding to PARP-1, which subsequently inhibited the expression of downstream pathways and related cytokines. This study provides a foundation and direction for the development and exploration of potential lead compounds for anti-hepatic fibrosis therapy (Huang et al. 2023).

### Cardioprotective activity

AMDis a promising medicinal drug for the treatment of diabetes and cardiovascular disorders due to its exceptional anti-lipid peroxidation effectiveness. Using phenylephrine, researchers in this work created the cardiac hypertrophy cell model in rat neonatal ventricular cardiomyocytes (RNVMs). The cell model exhibited elevated protein synthesis and cardiomyocyte size, which can be restored with AMD administration in a manner that is dependent on both concentration and time. The administration of AMD improved the impairment of cardiac function and alleviated the cardiac hypertrophy indicators, such as ventricular mass/body weight, myocyte cross-sectional area, and the expression of ANP, BNP, and Myh7, in the transverse aortic constriction (TAC) induced cardiac hypertrophy model. Treatment with AMD also reduced heart fibrosis and the associated fibrogenic genes. Subsequent research showed that AMD may reverse the effects of pressure overload on the expression of 4-HNE and malondialdehyde (MDA), restore the reduction in the GSH/GSSG ratio, and stop the nuclear translocation of NFAT and the activation of MAPK pathways. In conclusion, AMD protects mice against experimental cardiac hypertrophy, indicating that it may one day be used as a novel therapeutic medication to treat pathological cardiac hypertrophy (Cui et al. 2018).

### Anti-acetylcholinesterase activity

When studying Biologically active flavonoids from *Dodonaea viscosa* and investigated their antioxidant and anti-cholinesterase activity. AMD among the isolated compounds had inhibitory activity against AChE (IC_50_ = 173.22 µM) and BChE (IC_50_ = 95.13 µM) (Muhammad et al. 2015). In another study, The antioxidant, and cholinesterase-inhibiting properties of ethanol extracts from five Origanum species *O. majorana* L., *O. onites* L., *O. syriacum* L., *O. vulgare* subsp. hirtum (Link) Ietsw., and *O. vulgare* subsp. viride (Boiss.) Hayek—that were collected yearly (every month) were examined for their cholinesterase inhibition and antioxidant activity. AMD which was tentatively identified in LC-HRMS/MS metabolite profiling of *Origanum* spp. Was also investigated for these activities. Correlation coefficients between the tentatively annotated AMD (absolute peak areas) and bioactivity results IC_50_ values are for AChE, BChE, Metal-chelation, FRAP and DPPH radical scavenging; 0.514, 0.445, 0.202, 0.367 and 0.011μg/mL, respectively. Significant correlations were observed between AMD and AChE, BChE, and FRAP results (Gök et al. 2022).

### Cytotoxicity

Upon screening the cell viability of fractions and flavonoids including AMD which were isolated from *Brugmansia arborea* L. flowers, Kim et al. tested their effect on RAW 264.7 cell viability using an MTT assay. It was found that All fractions and compounds showed no cytotoxicity at concentrations lower than 100 μg/mL (Kim, Jang, Jung, Oh, Oh, Lee, Kang, Kim, Lee, Baek and Biotechnology 2020). In a separate study, researchers successfully isolated AMD and trans taxifolin from *Opuntia humifusa*. Interestingly, DHK exhibited synergistic effects when combined with taxifolin, leading to the inhibition of HeLa cell xenograft tumor growth. Moreover, *O. humifusa* extracts demonstrated the ability to induce a G1 phase arrest in HeLa cells. This was evidenced by a decrease in cell cycle regulators such as Cdk4, as well as a reduction in levels of cyclin D1 and phosphorylated Rb proteins associated with the G1 phase. Flow cytometric analysis revealed an increase in the number of cells in the G1 phase, corresponding to the observed inhibition ratio (Zou et al. 2005). Their synergistic effects and ability to induce G1 phase arrest in HeLa cells hold promise for future therapeutic applications. Further investigations are necessary to elucidate the underlying mechanisms and explore the clinical implications of these compounds in cancer treatment.

AMD, which was isolated from the methanol extract of the capitula of Coreopsis tinctoria Nutt. (Asteraceae), has been found to possess inhibitory activity towards aromatase enzyme. This inhibition is competitive in nature and is relatively potent, with an inhibition rate of 0.32 μM. Its potent inhibitory activity can be attributed to the presence of a 3',5'-resorcinol moiety at the B ring in its flavanone skeleton, which is shared by other known aromatase inhibitors such as aminoglutethimide (0.84 μM) and chrysin (0.23 μM). Moreover, the active constituent of AMD specifically targets aromatase and does not inhibit the activity of the 5α-reductase enzyme, which has the same substrate "testosterone." These findings suggest the potential specificity of AMD as an aromatase inhibitor (Luo et al. 2023).

### Anti-viral activity

Since December 2019, the world has been grappling with the COVID-19 pandemic caused by the severe acute respiratory syndrome coronavirus 2 (SARS-CoV-2). While vaccines have been developed to prevent infection, effective medicines are still needed to control the virus. Human coronavirus 229E (HCoV-229E) is responsible for the common cold. The main protease (Mpro) plays a crucial role in the replication of both viruses within host cells, making it an ideal target for screening potential medicinal compounds. In this study, researchers conducted docking simulations with two Mpro enzymes and AMD. Additionally, they investigated the in vitro inhibition of SARS-CoV-2 Mpro and the replication of HCoV-229E. The docking simulation results indicated that compound could bind to multiple subsites in the Mpro binding pocket (S1, S1', S2, and S4) and inhibit SARS-CoV-2 Mpro activity. Further in vitro inhibition assays demonstrated the compound effectively inhibited SARS-CoV-2 Mpro activity, with IC_50_ values of 20.3 μM. These findings highlight the potential of these antioxidative dihydroflavonols as promising candidates for combating both viruses (Zhu et al. 2022).

### Anti-apoptotic activity

The administration of AMD exhibited a substantial increase in the levels of cleaved caspase-3, caspase-9 fragments, and cleaved PARP. This effect was achieved by upregulating the expression of Bax and Bad while downregulating the expression of Bcl-2 and Bcl-xL. Consequently, dihydrokaempferol effectively initiated cell death in the target cells. AMD, distinguished by a hydrogenated double bond between C-2 and C-3 compared to kaempferol, demonstrated potent anti-proliferative and pro-apoptotic properties (Zhang et al. 2018). Previous investigations have shown that kaempferol inhibits the proliferation and migration of human fibroblast-like synoviocytes, as well as reducing the severity of arthritis and osteoclastogenesis (Singla et al. 2023). In this study, AMD exhibited a strong anti-proliferative and pro-apoptotic impact. This effect was mediated through the upregulation of Bax and Bad expression, the downregulation of Bcl-2 and Bcl-xL expression, and the initiation of the caspase cascade, ultimately leading to cell death (Pan et al. 2018). Based on these findings, AMD holds promise as a potential therapeutic option for rheumatoid arthritis (RA). The mechanism by which dihydrokaempferol promotes apoptosis in RA fibroblast-like synoviocytes (RA-FLSs) is associated with the caspase-dependent mitochondrial signaling pathway. Further research is necessary to fully elucidate the underlying mechanisms and to explore the clinical implications of dihydrokaempferol in the treatment of RA.

## Some important derivatives of AMD and their biological activities

Dihydrokaempferol-3-*O*-α-L-rhamnoside derived from *Cupressus macrocarpa* methanol extract exhibited promising antibacterial activity, against the *Staphylococcus enterica* isolated with MIC value of range 64–256 ug/mL (Elmongy et al. 2022).

Furthermore, (2R,3S)-Dihydrokaempferol 3-*O*-β-D-glucoside and (2S,3R)-dihydrokaempferol, and 3-O-β-D-glucoside, isolated from *Agrimonia pilosa* Ledeb. The compounds showed inhibitory anti-AChE activity at concentrations of 4, 20, and 100 μM. In comparison to (2S,3R)- compound, (2R,3S)- showed weak inhibitory activity. This was due to the position of β-D-glucose at C-40, C-3, and C-7 in taxifolin glucoside that did not cause any inhibitory effect (Seo et al. 2017). Additionally, through structure–activity relationship (SAR) analysis, it was determined that out of the six tested compounds, (2R,3R) -(+)-dihydrokaempferol-7,4'-dimethylether and eriodictyol-7,4'-dimethylether displayed the highest antiproliferative activity. This potency could be attributed to the presence of methoxy groups and a single bond at the C2-C3 position of these compounds (Hahm et al. 2015).

The gastroprotective properties of aromadendrin-4'-*O*-methyl-ether were investigated in mouse models of ethanol/HCl- and indomethacin-induced gastric ulcers. Various histological, histochemical, oxidative, and inflammatory parameters were analyzed in the ulcerated tissues. Acid antisecretory activities were also assessed. The minimum effective oral dose of aromadendrin-4'-*O*-methyl-ether for gastroprotection was determined to be 3 mg/kg. This dose was found to normalize the activities of superoxide dismutase, catalase, and glutathione-S-transferase, which are important antioxidant enzymes. Additionally, it led to a reduction in myeloperoxidase activity, indicating a decrease in inflammatory response at the ulcer site.

Furthermore, aromadendrin-4'-*O*-methyl-ether was observed to increase the gastric mucin content, which plays a crucial role in maintaining the protective mucosal barrier of the stomach. It also significantly decreased the volume, pH, total acidity, and pepsin activity of gastric juice in rats, suggesting a reduction in gastric acid secretion (Costa et al. 2018).

## Conclusions and future perspectives

Extensive research has highlighted the significant potential of AMD in the prevention and management of chronic illnesses, including cancer, inflammatory conditions, oxidative stress, and hyperglycemia. The clinical studies of AMD for hyperinsulinism, insulin resistance, and hyperglycemia could be of interest for upcoming research. Furthermore, researchers have explored the hepatoprotective, cardioprotective, and neuroprotective activities of AMD, shedding light on its putative mechanism of action. However, it is crucial to establish a criterion to overcome any potential cytotoxic effects associated with AMD. Moreover, it is worth noting that AMDexhibits low oral bioavailability, necessitating the reliance on nanocarriers for its current applications. Further research and clinical studies are warranted to explore their therapeutic potential and pave the way for potential drug candidates in the fight against these viral infections.

To enhance its bioavailability, the combination of polysaccharides with oligosaccharide complexes has shown promise. Furthermore, further research is imperative in the realm of food science to ascertain the potential hazards of high AMD intake. While AMD holds remarkable therapeutic potential, it is essential to carefully assess the dosage and potential negative side effects associated with its consumption in larger quantities. By delving into these crucial aspects, researchers aim to unlock the full potential of AMD and ensure its safe and effective utilization in combating chronic diseases. With ongoing investigations, we can pave the way for harnessing the therapeutic benefits of this remarkable compound while mitigating any potential risks.

## Data Availability

Not applicable.
